# Barriers and enablers affecting female participation in physical activity

**DOI:** 10.3389/fgwh.2025.1707792

**Published:** 2026-01-16

**Authors:** Ashraf A’aqoulah, Wafaa Alonazi, Farah Kalmey, Nisreen Innab, Raghib Abusaris

**Affiliations:** 1 Health Systems Management Department, College of Public Health and Health Informatics, King Saud bin Abdulaziz University for Health Sciences, Riyadh, Saudi Arabia; 2 King Abdullah International Medical Research Centre, Riyadh, Saudi Arabia; 3 Department of English Language, College of Science and Health Professions, King Saud bin Abdulaziz University for Health Sciences, Riyadh, Saudi Arabia; 4 Department of Computer Science and Information Systems, College of Applied Sciences, AlMaarefa University, Riyadh, Saudi Arabia; 5 Epidemiology and Biostatistics Department, College of Public Health and Health Informatics, King Saud bin Abdulaziz University for Health Sciences, Riyadh, Saudi Arabia

**Keywords:** fitness, health system policymaker, physical activity, Saudi Arabia, Saudi women, women's health

## Abstract

**Background:**

Physical activity offers numerous health benefits and helps prevent various diseases, making it an essential component of a healthy lifestyle.

**Objective:**

This study aims to examine the barriers and enablers that affect female participation in physical activity.

**Methods:**

This was a cross-sectional quantitative study. The study questionnaire was adopted from a previous study. The survey was conducted online and completed by 668 Saudi women from across the country.

**Results:**

The study findings regarding low physical activity levels among Saudi women are concerning, as 72.2% of participants were classified as sedentary or physically inactive. The study revealed that age, employment status, and income were significant factors affecting engagement of women in physical activity. Moreover, barriers such as expensive gym memberships, a lack of women-only clubs, a lack of enjoyment in sports, and prolonged use of the same exercise devices prevented Saudi women from participating in physical activities. However, women reported exercising to boost their self-satisfaction and self-confidence, thereby promoting overall health.

**Conclusion:**

Saudi women exhibit a low level of physical activity. Barriers such as expensive gym memberships, a lack of women-only clubs, a lack of enjoyment in sports, prolonged use of the same exercise devices, and motivations related to self-satisfaction and self-confidence prevent Saudi women from practicing physical activities. Health system policymakers need to take action to increase physical activity levels and address these barriers.

## Introduction

Physical activity is an important aspect of a healthy lifestyle. It involves bodily movement resulting from skeletal muscle contractions that increase energy expenditure above resting levels. Physical activity is associated with a reduced risk of non-communicable diseases, such as diabetes and obesity (1). In addition, physical activity provides numerous health benefits and contributes to disease prevention. It also helps in the management of various health conditions. However, studies have shown that most Saudi Arabian women do not engage in sufficient physical activity, which is concerning (2, 3). In Saudi Arabia, approximately 60% of the population is considered physically inactive (1).

Lifestyle factors, sociodemographic characteristics, and the unwillingness of Saudi women to engage in physical activity have led to an increase in non-communicable diseases. Lifestyle practices such as fast-food intake and consumption of dairy products and milk as well as sociodemographic factors including age, income, and education level are major contributors to obesity and overweight among Saudi women. Most obese women in Saudi Arabia were married, had lower education levels, belonged to low-income groups, had a greater number of obese family members, and were elderly (4).

Based on the social ecological model (SEM), female behavior is influenced by multiple factors, such as individual factors (motivation and confidence), interpersonal factors (family support and cultural expectations), organizational factors (work hours and gym access), community factors (availability of safe walking areas), and policy-level factors (national initiatives and women-only gyms). Several factors have been identified as causes of physical inactivity. Foremost among these is the attitude of Saudi women toward physical activities, which has been identified as a contributing factor to physical inactivity. A positive attitude is crucial for engaging in activities that improve quality of life and reduce the likelihood of developing some chronic diseases related to lifestyle factors. Al-Haramlah et al. examined attitudes of Saudi women toward physical activity based on their province, age, weight, education level, and occupation and confirmed that these factors contribute to low levels of physical activity. Their findings showed that positive attitudes toward physical activity provide immeasurable benefits (5). However, these benefits were limited by factors such as age, marital status, education level, occupation, weight, and place of residence. Alahmadi and Almasoud assessed four components of physical fitness to determine the level of physical fitness. Their results indicated an alarming lack of physical fitness, characterized by high body fat percentage, poor muscle strength, low flexibility, and low cardiorespiratory fitness (6).

Sports activities are a great way for women to stay physically active. Aldegheiry (2021) mentioned that sports activities and use have a positive impact on the fitness and psychological well-being of Saudi women. In addition, sports participation has been found to have a significant impact on both mental and social aspects (7). However, studies indicate that Saudi women often do not participate in sports due to financial barriers to joining sports clubs and a lack of awareness of the benefits of physical activity (8). Furthermore, the long-standing absence of physical education in girls’ schools in Saudi Arabia has contributed to a predominantly sedentary lifestyle among women, resulting in a lack of motivation to engage in physical activity (9). Samara et al. and Sunarti confirmed that women demonstrate a strong willingness to engage in gym-based physical activity and that motivation for physical education among women has increased. Limited access to fitness centers and insufficient motivation contributed to low physical activity among women (10, 11).

Therefore, it is necessary to raise awareness among women about the benefits of physical activity and exercise to promote population health. Sports have a significant positive impact on both individual and societal health (7, 12). A healthy society cannot exist without healthy members; therefore, promoting health should be a national priority. The Saudi Arabian government should encourage the participation of women in sports activities to achieve better health outcomes. The future development of a country depends on the contributions of all its citizens. Therefore, this study aims to examine the associations between demographic variables (age, marital status, region, education level, employment status, and income), physical activity barriers (such as lack of time for physical exercise and the high cost of gym memberships), physical activity enablers (such as boosting self-satisfaction, self-confidence, and keeping the body fit and healthy), and physical activity engagement among Saudi women.

## Methods

This cross-sectional quantitative study targeted all Saudi women aged 18 years and older living in the Kingdom of Saudi Arabia. The study was conducted online and included participants from all five regions of the country. Data were collected during May, June, and July of 2022. The sample size was 668 participants who completed the survey.

### Sampling method

A convenience sampling technique was used in this study. The physical activity survey, adapted from a previous study questionnaire, was administered online using Google Forms and distributed to the target population through various social media applications, including Telegram, Twitter, WhatsApp, Instagram, and LinkedIn. All questions were marked as obligatory to ensure that none were left unanswered. Participants were directed automatically to the next section once all the questions had been answered. The inclusion criteria required participants to be female and at least 18 years old. The exclusion criteria included males and females under 18 years.

### Data collection tool

The validated and reliable tool used in this study was adopted from a previous study (7). The survey tool was developed to assess barriers to and enablers of physically active behavior among Saudi women. The Brislin back-translation method was used. The questionnaire contained three main sections. The first section collected demographic information, such as age, marital status, region, education level, and employment status, with varying response options. The second section assessed barriers to physical activity, such as the high cost of gym membership, limited or unavailable public walking places, and the lack of women-only clubs. The third section evaluated enablers of physical activity, such as maintaining physical health, boosting self-satisfaction and self-confidence, viewing sports activity as an investment of time (as physical activity requires women to spend time on it), reducing anxiety and stress, and promoting mental health. For the second and third sections, response options were limited to yes or no. In addition, the questionnaire included items that asked participants to report the number of minutes they spent exercising per week. According to World Health Organization guidelines, adults are considered physically active if they engage in 150–300 min of moderate-intensity physical activity per week or 75–150 min of vigorous-intensity physical activity per week (13).

### Data analysis

The study data were analyzed using the Statistical Package for the Social Sciences (SPSS). Categorical variables were summarized using counts and percentages. In addition, the chi-square test (*χ*^2^) was used to examine associations between physical activity levels of women and demographic characteristics, enablers, and barriers. Furthermore, to assess the strength of association, multivariable binary logistic regression analyses were utilized. Statistical significance was set at a *P*-value ≤ 0.05.

## Results

### Demographic variables

Table 1 demonstrates that the majority of participants were aged 18–28 years (76.6%), which indicates a predominantly young sample. The majority of participants were single (79.6%), resided in the central region (49.6%), and held a bachelor’s degree (71.0%). In addition, more than half of the participants were students (53.6%) and most of them earned less than SAR 5,000 (67.8%).

**Table 1:** Descriptive statistics of demographic data.

Item	Category	No. (%)
Age group (years)	18–27	512 (76.6)
	28–37	90 (13.5)
	38–47	41 (6.1)
	48–57	22 (3.3)
	58 or above	3 (0.4)
Marital status	Single	532 (79.6)
	Married	115 (17.2)
	Separated	20 (3)
	Widowed	1 (0.1)
Region	Central	331 (49.6)
	Western	130 (19.5)
	Southern	47 (7)
	Eastern	104 (15.6)
	Northern	56 (8.4)
Education level	Secondary school or less	139 (20.8)
	Undergraduate	474 (71)
	Postgraduate	55 (8.2)
Employment status	Employed	195 (29.2)
	Not working	115 (17.2)
	Student	358 (53.6)
Monthly household income	SAR 20,000 or more	28 (4.2)
	SAR 10,000–less than SAR 20,000	81 (12.1)
	SAR 5,000–less than SAR 10,000	106 (15.9)
	Less than SAR 5,000	453 (67.8)

**Figure 1:**
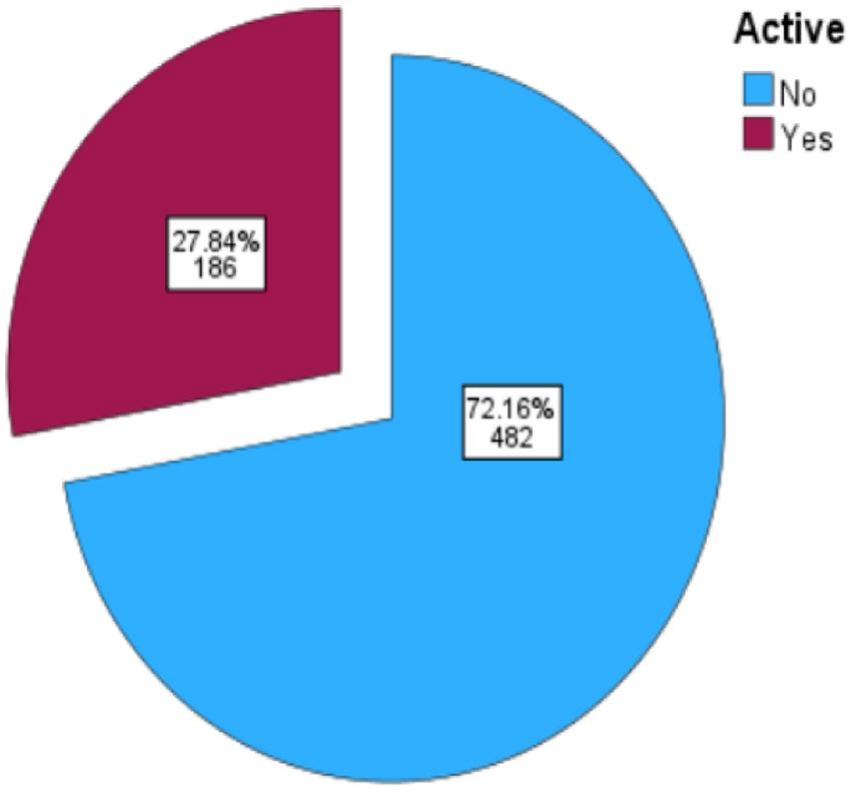
Percentages of physically active and inactive participants.

Based on the WHO definition of physical activity/inactivity, and as shown in Figure 1, the study revealed that 72.2% of participants were physically inactive (95% CI: 68.6%–75.5%).

### Physical activity and demographic characteristics

Table 2 examines the association between demographic variables and physical activity levels of women. Unlike region and marital status, age, education level, monthly income, and employment status were significantly associated with the physical activity of women.

**Table 2:** Association between demographic variables and physical activity levels of women.

Variable	Category	Total	Physically active no. (%)	Physically inactive no. (%)	*P*-value
Age (years)	18–27	512	134 (26.2)	378 (73.8)	**0.02** *
	28–37	90	24 (26.7)	66 (73.3)	
	38–47	41	14 (34.1)	27 (65.9)	
	48–57	22	12 (54.5)	10 (45.5)	
	Above 58	3	2 (66.7)	1 (33.3)	
Marital status	Single	532	153 (26.9)	389 (73.1)	0.273^a^
	Married	115	34 (29.6)	81 (70.4)	
	Separated	20	8 (45.0)	12 (55.0)	
	Widowed	1	0 (0.0)	1 (100.0)	
Region	Central	331	97 (29.3)	234 (70.7)	0.931
	Western	130	35 (26.9)	95 (73.1)	
	Southern	47	13 (27.7)	34 (72.3)	
	Eastern	104	26 (25.0)	78 (75.0)	
	Northern	56	15 (26.8)	41 (73.2)	
Education level	High school or less	139	31 (22.3)	108 (77.7)	**0.005** *
	Bachelor's degree	474	130 (27.4)	344 (72.6)	
	Postgraduate	55	25 (45.58)	30 (54.5)	
Employment status	Employed	195	66 (33.8)	129 (66.2)	**0.015** *
	Unemployed	115	37 (32.2)	78 (67.8)	
	Student	358	83 (23.2)	275 (76.8)	
Monthly income	SAR 20,000 or more	28	13 (46.4)	15 (53.6)	**0.013** *
	SAR 10,000–less than SAR 20,000	81	25 (30.9.6)	56 (69.1)	
	SAR 5,000–less than SAR 10,000	106	30 (28.3)	76 (71.7)	
	Less than SAR 5,000	453	116 (25.6)	337 (74.4)	

* *P*-value is significant at the level *P* ≤ 0.05.

a Using the Fisher–Freeman–Halton's exact test.

By employing multivariable binary logistic regression, Table 3 depicts the demographic predictors of physical activity levels of women. The odds of being physically active among women aged 28–37 years decreased by approximately 47% compared to those aged 18–27 years. Likewise, compared to employed people, the odds of being physically active were approximately 46% lower among unemployed women and 67% lower among students. Similarly, compared with those who earn less than SAR 5,000, the odds of being physically active decreased by 46% among those earning SAR 5,000–10,000 and by 50% among those earning SAR 10,000–20,000.

**Table 3:** Demographic predictors of physical activity engagement of women.

Variable	Category	Coefficient	*P*-value	aOR	OR 95% CI	
					Lower	Upper
Age (ref. 18–27 years)	28–37	−0.637	**0.** **037** *	0.529	0.291	0.961
	38–47	−0.221	0.572	0.801	0.372	1.728
	48–57	0.764	0.120	2.146	0.819	5.623
	58 or above	1.583	0.209	4.872	0.412	57.585
Education (ref. ≤HS)	Bachelor	−0.092	0.625	0.912	0.631	1.318
	Postgraduate	0.685	0.071	1.984	0.942	4.178
Employment (ref. employed)	Unemployed	−0.613	**0.** **016** *	0.542	0.330	0.891
	Student	−1.112	**<0** **.** **001** *	0.329	0.235	0.461
Income (ref. <5,000)	5,000 > 1,000	−0.620	**0.** **031** *	0.538	0.306	0.944
	1,000 > 20,000	−0.701	**0.** **038** *	0.496	0.256	0.963
	≥20,000	0.388	0.400	1.474	0.597	3.641

Hosmer–Lemeshow test's *P*-value = 0.880.

* *P*-value is significant at the level *P* ≤ 0.05.

### Barriers and enablers associated with physical activity

Table 4 shows that barriers like expensive gym memberships, lack of women-only clubs in the participants' areas, loss of enjoying playing sports, prolonged use of the same exercise devices, caring for a family member (child or adult), other more important responsibilities, and lack of time for exercise were significantly associated with weekly sports participation of women in Saudi Arabia (*P* < 0.05). On the other hand, enablers such as exercising to maintain physical health, staying fit, boosting self-satisfaction and self-confidence, and perceiving sports as worthwhile were significantly associated with weekly sports participation of women (*P* < 0.05).

**Table 4:** Significant barriers to and enablers of physical activity of women.

Doma	Variable	Category	Total	Physically inactive count (%)	Physically active count (%)	*P*-value
Barriers	Gym memberships are expensive	No	122	76 (62.3)	46 (37.7)	**0.** **007** *
		Yes	546	406 (74.4)	140 (25.6)	
	Lack of women-only clubs in my area	No	348	236 (67.8)	112 (32.2)	**0.** **009** *
		Yes	320	246 (76.9)	74 (23.1)	
	Do n't enjoy playing sports	No	582	401 (68.9)	181 (31.1)	**<0** **.** **001** *
		Yes	86	81 (94.2)	5 (5.8)	
	Prolonged use of the same exercise devices because of lack of knowledge to use other devices	No	483	329 (68.1)	154 (31.9)	**<0** **.** **001** *
		Yes	185	153 (82.7)	32 (17.3)	
	Caring for a family member (child or adult)	No	550	388 (70.5)	162 (29.5)	**0.** **045** *
		Yes	118	94 (79.7)	24 (20.3)	
	Not important compared to other responsibilities	No	595	422 (70.9)	173 (20.1)	**0.** **043** *
		Yes	73	60 (82.2)	13 (17.8)	
	Do n't have time to exercise	No	376	232 (61.7)	144 (38.3)	**<0** **.** **001** *
		Yes	292	250 (85.6)	42 (14.4)	
Enablers	Maintain physical health	No	54	49	5	**0.** **001** *
		Yes	614	433 (70.5)	181 (29.5)	
	Keeping the body fit and healthy	No	56	50 (89.3)	6 (10.7)	**0.** **003** *
		Yes	612	432 (70.6)	180 (29.4)	
	Boost self-satisfaction and self-confidence	No	81	77 (95.1)	4 (4.9)	**<0** **.** **001** *
		Yes	587	405 (69.0)	182 (31.0)	
	Physical activity is important	No	93	76 (81.7)	17 (18.3)	**0.** **027** *
		Yes	575	406 (70.6)	169 (29.4)	

* *P*-value is significant at the level *P* ≤ 0.05.

Utilizing multivariable binary logistic regression, Table 5 reveals that “boosting self-satisfaction and self-confidence” was the only enabler that remained significantly associated with an increased likelihood of physical activity (OR = 2.266), whereas barriers including “expensive gym memberships, lack of women-only clubs in participants' areas, lack of enjoyment in sports, and loss of interest due to prolonged use of the same exercise devices” remained significantly associated with a decreased likelihood of physical activity (OR = 0.535, 0.632, 0.171, and 0.571, respectively).

**Table 5:** Barriers and enablers as predictors of physical activity of women.

Variable	Coefficient	*P*-value	aOR	OR 95% CI	
				Lower	Upper
Maintain physical health	0.213	0.604	1.237	0.554	2.765
Keep the body fit	−0.090	0.822	0.914	0.417	2.002
Boost self-satisfaction and self-confidence	0.818	**0.** **016** *	2.266	1.166	4.403
Physical activity is important	−0.410	0.161	0.664	0.374	1.177
Expensive gym membership	−0.626	**0.** **004** *	0.535	0.348	0.822
Few or no public places to walk	0.128	0.505	1.136	0.781	1.653
Lack of women-only clubs	−0.459	**0.** **017** *	0.632	0.433	0.921
Lack of enjoyment in sports	−1.768	**<0** **.** **001** *	0.171	0.066	0.443
Prolonged use of the same exercise devices	−0.560	**0.** **018** *	0.571	0.359	0.909

Ref. = No; Hosmer–Lemeshow test's *P*-value = 0.880.

* *P*-value is significant at the level *P* ≤ 0.05.

## Discussion

The study's findings regarding low physical activity levels among Saudi women are concerning, as 72.2% of participants were classified as sedentary or physically inactive. This is particularly alarming given the increasing prevalence of lifestyle-related diseases, such as heart disease, obesity, and diabetes, in the country. Alsulaiman and Abu-Saris reported that sedentary behavior was strongly associated with depression, anxiety, and stress (14). The study highlights the need for interventions to promote physical activity among Saudi women.

The results revealed a positive correlation between age and physical activity engagement among women in Saudi Arabia. Women aged 18–27 years were physically active more than those aged 28–37 years. This result is consistent with a study conducted by Hammer, which found that younger women are more physically active than older women (15). This could be because younger women frequently have more flexible exercise schedules and fewer caregiving duties.

The study found a positive correlation between employment status and physical activity engagement among women in Saudi Arabia, suggesting that employed women have a greater probability of engaging in physical activity than those who are unemployed. These findings are consistent with studies by Nabil and Answer and Almalki et al., which found employment status and education to be significant predictors of physical activity engagement (16, 17). This result may be attributed to the greater financial independence of working women, enabling them to afford gym memberships, transportation to fitness facilities, or participation in structured programs. In contrast, non-working women may rely on family support, which can limit their options.

Similarly, women earning less than SAR 5,000 were more likely to engage in physical activity than those earning more than SAR 5,000. This result is consistent with the study by Pan, which reported that, overall, individuals with lower incomes tend to be more physically active than those with higher incomes (18). This pattern may be explained by the fact that lower-income women typically accumulate physical activity through occupational work and household tasks rather than solely through gym attendance.

The study found a positive correlation between enhanced self-satisfaction and self-confidence and physical activity engagement among women in Saudi Arabia. It revealed that women who reported high levels of self-satisfaction and self-confidence were more likely to engage in physical activity than those who did not. These findings are consistent with those of Twardowska et al., who reported similar results (19). This result may be explained by the fact that physical activity can improve satisfaction with one's appearance, which enhances feelings of competence and self-worth. Nie et al. reported a positive association between physical activity and satisfaction, which contributes to enhanced self-esteem (20).

The study also identified a negative association between expensive gym membership and physical activity engagement. This means that women who reported that gym memberships are expensive were less likely to engage in physical activity. This result is consistent with the study by AlSayegh, which reported a similar result (21). This association may be explained by the fact that many women in Saudi Arabia are not employed. Consequently, non-working women often rely on family support, which can limit their ability to join a gym (22).

Another finding identified as a barrier in this study is the lack of women-only clubs. Women who reported a lack of women-only clubs were less likely to engage in physical activity. This result is consistent with a study by Mann and Hacker, which reported that the lack of clubs affects the engagement of girls and women in sports. This association may be explained by cultural norms that require women to attend women-only clubs. In addition, women may feel the need to maintain their privacy. A study reported that women prefer gyms with female staff and female users to maintain personal privacy (23).

Additionally, the study found a negative association between enjoyment in sports and engagement in physical activity. Women who reported not enjoying sports were less likely to engage in physical activity. This result is consistent with findings from other studies (16, 24). Similarly, Snuggs et al. and Blackford et al. reported that the need to maintain proper health and finding enjoyment in sports are key enabling factors for engagement of women in physical activities (25, 26). Limited encouragement or criticism of girls during childhood may reduce their enjoyment in sports and decrease their likelihood of engaging in physical activity later in life (27).

Finally, the study found that women who reported prolonged use of the same exercise devices were less likely to engage in physical activity. This result is consistent with the study by Deelen et al., which reported that individuals who use a variety of fitness equipment and practice different exercise techniques are more likely to enjoy engagement in gym-based physical activity. This may be because variety in equipment and techniques helps prevent boredom while working out at sports gyms.

Therefore, health system policymakers need to take action to encourage women to engage in physical activities by conducting public awareness campaigns to educate women about the importance of physical activity and its benefits for overall health, including its role in enhancing self-satisfaction and self-confidence, establishing low-cost community centers and free public recreational spaces that may help reduce financial barriers, encouraging the opening of women-only sports clubs in different areas, encouraging girls to practice sports during childhood, and increasing awareness regarding using a variety of equipment and techniques.

## Limitations of the study

A limitation of the study is the lack of previous research on physical activity among Saudi women, which restricts the ability to compare the current findings with those from existing studies, which can limit the overall understanding of the context and implications of the results. Moreover, the cross-sectional design of the study prevents the establishment of a causal relationship between the identified factors and physical activity engagement among Saudi women. In addition, it is challenging to generalize the results to other communities. Also, other aspects not included in the analysis may limit the scope of this study. Furthermore, the sampling method is convenience sampling.

## Conclusion

Physical activity is associated with numerous health benefits and helps prevent various diseases. Physical activity is a critical component of a healthy lifestyle. This study examined the association between different demographic variables and physical activity engagement among women in Saudi Arabia. It also investigated the barriers to and enablers of physical activity and their association with physical activity levels. The study revealed that most Saudi women were physically inactive. The results demonstrated significant associations between the variables and physical activity. The results show that boosting self-satisfaction and self-confidence, expensive gym memberships, the lack of women-only clubs, the lack of enjoyment in sports, and the prolonged use of the same exercise devices were barriers that prevent Saudi women from practicing physical activities. Further studies are needed to examine the causal relationships between the identified factors and physical activity engagement among Saudi women. In addition, future studies should employ random sampling techniques.

## Data Availability

The raw data supporting the conclusions of this article will be made available by the authors, without undue reservation.
